# The Beginnings of Education

**Published:** 1915-09

**Authors:** Henry Jones Mulford

**Affiliations:** Buffalo, N. Y.; 143 Allen Street


### The Beginnings of Education.

Some Suggestions to Parents and Teachers.

By HENRY JONES MULFORD, M. D., Buffalo, N. Y.

Second Paper—The Teacher.

Teaching belongs to the gentle arts; for, if gentleness counts for anything at all in this world here is the place where it counts for the most. But, teaching has been anything but gentle. The teacher has been a "driver;" he has held the reins and the one at the farther end has gone as the driver directed. And, usually, the driver has held the reins in one hand and a whip in the other.

To drive! What a way to teach; what a way to teach young children! Pedagogy so construed, so misconstrued, becomes for the child just a monotony of days. Day after day the same round, a vicious circle; a circle encompassed by fear and fatigue. What a way to teach! Is it any wonder that man has been so long in "arriving?" As some one has said: "Progress to man seems rather to happen than to be achieved."

But we begin to realize the possibilities of teaching; we realize that the teacher must guide rather than drive. And we have discovered where the child belongs in the human scale. We find that the child is only a child, that he has only a child brain, a brain bound by the limitations of the era of its development. Knowing this we are able to perceive the limitations of the child mind. We find that a pint cup will hold only a pint; and we find, also, that there are good and bad ways for putting things into even such an obvious thing as a pint cup.

What is teaching? In what does this "gentle art" consist? A definition will not be difficult if we recall what was said in the previous paper. Teaching is the gentle art of developing the conscious memory through action without fatigue. Through the art of the teacher the cells of the higher brain centres are developed; but no fatigue should follow as a result of the process.

But haven't we seen that fatigue is a natural consequence of all effort, whether the effort be mental or muscular? And if natural how may it be avoided? Yes, fatigue is a natural outcome, but, fatigue as actual tiredness is never present except through unnatural circumstances. The beginning of fatigue attends all effort, but real fatigue comes only through over-effort. We see, then, that we cannot avoid the approach to fatigue, but we can avoid the actual production of fatigue. We must avoid the production of fatigue in the child brain, for, the child brain being a brain in the process of growth, is easily upset. And, if memory is the main factor in the development of the brain, and fatigue is the main factor in the retardation of that development, then it is quite obvious that fatigue is the one great thing to be avoided in the development of the child.

If memory is the main factor in brain development then it is to memory that we must look for success in that development. What is the best method for developing the memory? We lay the foundation of memory just as we lay the foundation of the cerebral cortex. The two, in fact, are laid simultaneously; that which influences the one influences the other. As the cerebral cortex has developed through action, then memory must develope through action. Action is at the bottom of the development of the brain cells, and the foundations of both substance and function are laid through that. If these foundations are laid properly in childhood the brain always will be a willing instrument. A brain that has developed in the right manner always will have good memory; and no man possessing such a brain ever will need to pursue that will-o'-the-wisp seen so often in the advertising pages of our magazines "Lost Memory Restored."

Is it possible to "restore" a lost memory? Speaking generally we can answer no. None of the "systems" in vogue can restore a lost memory or strengthen an impaired one. It may be that a "system" will enable one to fix a certain idea in the mind; but, if it does, it is only through a round-about way. And, even though it helps with the one idea, the being able to recall the one does not help with other ideas. The "system" does not improve the general faculty of memory, it merely fixes independent ideas more or less firmly in the mind through an unnecessarily laborious process. In order to recall each idea it is necessary to employ the same process for each. This puts a tremendous lot of extra work upon the brain cortex, and weakens, rather than strengthens the memory. The fact is, that memory cannot be influenced except through the fundamental process: the memory can be recreated only after the manner of its creation. If the memory fails in adult life in an otherwise normal individual it is because of its lack of training, or, of faulty training, in the youth of the individual. It is then impossible to restore it because the cerebral cortex has become fixed in the wrong way; and there is no "system" known that will alter the development of an already developed brain. I want to impress upon you the fact that memory must be developed; it cannot be cultivated.

In developing the child brain through memory we have to remember that our results are to come through natural channels, through the activities of the child. No ideas should be presented to young children except active ideas, ideas that are alive, ideas that are visible. The idea must be accompanied by an object. The young child cannot grasp an idea for which his mind does not hold an image. One may describe an elephant to a child over and over again, but, until he goes to the Zoo and sees an elephant and feeds him peanuts he cannot know what an elephant is. Until the image of the elephant is in his mind the word is meaningless. And, not only is this true of young children, but it is true, also, of children of any age. The child learns best after this manner because this is the manner in which his brain tissue has developed. The brain tissue having formed this habit of procedure always must be approached through this habit. The habit has come in a natural manner, through a slow growth, a growth without effort on the part of the cerebral cells; and, having come in that manner, no fatigue has accompanied the process. We gather, then, that in order to obtain the proper result the brain cortex, that is, the memory, must be developed in the natural manner, slowly and without fatigue. We must not force development of the child brain: we must not urge function upon it too early. The centres must not be put to serious work before they are ready, they must not be pushed beyond their strength.

In my first paper I said that I believed that no child under eight years of age should receive formal instruction. Formal instruction before that age would force the brain centres, centres unprepared for complete function. The centres would be prepared to receive and to store up sensory impressions, for the exercise of memory, but they would not be prepared for real work, for the action of thought. They could receive, but they could not give. At this early period our special attention must be given to the development of memory, for the development of memory is the development of cortex. After the cortex is formed real instruction may be begun.

But, while formal instruction is improper before the age of eight, some sort of instruction will be in order. If the child is developing his cerebral cells he must develope them properly. The cells need the discipline of guidance. The images which come to them should come in an orderly sequence and receive proper registration, for these images are the thought images, they are the basis of future thought, and, as such, must receive careful attention. We must guide the child's activities; his life is a life of action, and it is through this action, this life-action, that he is to be developed. His life-action is mostly play action: his world is a play world. We should, then, get at him through his play; we should oversee his play and direct it. If there is a house to be built with blocks one should sit down with the child, and, without appearing to do so, direct the building. You build with him, and, in the building, you show him how to build, how to place the blocks to some purpose. If the child were left to build by himself he would place the blocks in a haphazard manner, so that the walls of the house would be all askew and unstable. The child might, after repeated efforts, get the thing right, but it would be only after the effort had been repeated over and over again in this futile way. There would be a great deal of wasted effort, and through this wasted effort, through the reception of these many unnecessary and wrong impulses the brain cells suffer from fatigue and confusion. If at the beginning the sensory impulses are the correct ones there is no wasted effort, there is no fatigue, no confusion, and the impulses are received in the right manner, consequently the brain image, the idea, is registered clearly and **permanently.**

In developing the memory we are preparing the brain for the higher functions of thought and reason; and, in proceeding in that direction in an orderly and natural manner, we develope these higher functions to their highest efficiency, and without injury to the brain tissue. I cannot believe in any system of child training that takes young children and makes them to read, write, and spell, at four or five years of age. In a child with unusual brain development, in a genius, this may be permissable, but, in the average child it is not. One can **make** the human animal do almost anything, **but forcing is not the proper thing where brain tissue is concerned.** We may be able to raise cucumbers after the hot-house method but not children.

The modern school room presents the most unnatural environment in which a child can be placed; there is no environment that can produce fatigue in more ways, nor more harm fully, than that. Its capacity for harm is three-fold: through bad air, wrong posture, overteaching. Of these three we shall confine our attention here to the last. It is the fatigue produced through overteaching that is the greatest cause of failure in our school rooms. Overteaching is overcrowding: it is forcing the cerebral cells beyond their normal capacity. In increasing cell activity we are increasing the products of that activity; we are producing fatigue. And this in an individual sitting in a school room When a child sits down his heart beats less frequently, and the blood current becomes slower. In a slow blood current the products of cell activity are not carried off as rapidly as they are in a more rapid current. And, if added to the slowing of the current, there is an overproduction of the tissue waste, if the fatigue producing substances are produced faster than they can be removed, the result is disaster to the cells in which they are retained. If the child were running about these matters would be removed as fast as they were produced. The activity of the child would increase the action of his heart, the blood current would be accelerated, and the cells would be washed free before they became choked up. It is for this reason that a child can run about upon his feet for so long without feeling fatigue, while pure brain action without activity upsets him in half the time.

In the child suffering from the fatigue of overteaching the brain is unable to retain the images coming to it; it repels the image-impulses through sheer inability to control them. The child then becomes inattentive and careless. When this happens it is a sign that the child needs a change; his thought centres have become fatigued, and the school room has become distasteful to him. He needs to have his blood current increased that his cells may be revived. Put the child out-of-doors for a half hour and note the change. He will become revivified; his cheeks will glow and his eyes sparkle under the renewed activity of the life current; and, what is more, his brain will be clear and ready for work.

The remedy for this sort of fatigue is **prevention**; it should not be allowed to assert itself. How is that to be brought about? As the cause lies in the modern school room then it is to that we must give our attention. The modern school room must be effaced; that is, it should be rearranged until it no longer resembles a school room. It should become a room in which there is no restraint, no tension, no fatigue: it should be a room in which there is a complete sense of mental freedom. To that end the fixed seat and desk must be removed, and the child should be permitted to move about freely. He may be permitted to do anything within reason to relieve strain upon his muscles and his brain cells. The school room should be made interesting and free; the number of pupils in one room should be reduced to ten; and the subjects for study must be so presented that the child mind will grasp them without effort.

There are few of us who enjoy eating a piece of dry bread; but, if that piece of bread be toasted and buttered, how it will be relished! It is the same with facts. A dry fact may be so dressed up, so toasted, made so crisp, that the brain will seize upon it and store it away without effort. It is effort, undue effort, that produces brain fatigue, and obstructs thought. Thought is just the calling up of successive images having a common relationship. The child will learn to think in a rational manner, therefore, if the images which go to make up his thought are assembled in the same manner.

We see, then, that teaching is not the simple thing it seems to be; we cannot just throw a fact at a child and force his brain to store it up. In fact we must not throw it at him at all. The forcing process then becomes too obvious and the fact is rejected. The reception of the fact must be through such a method that it will seem a pleasure rather than a duty. **The**

**fact must be alive, must be active: it is action that appeals to action.** In my first paper I called attention to the fact that the primitive brain was a brain of action, and, I said that the child brain was a primitive brain. I repeat that here because I want to impress upon you the fact that **action is the basis of all brain function.** In man this covers a broader scope than it does in the other animals, for it influences the higher centres as well as the lower. Observers are agreed upon this point. Professor James, late professor of Psychology at Harvard, said: ''All consciousness is motion.'' Herbert Spencer: ''Play is the child of work.'' Henry Drummond: ''The first law of evolution is motion.'' Someone else: ''To keep boys contented things must move.'' Another: ''An idea always tends to work itself out in action.'' And another: ''If man is made of dust, the boy is dust plus electricity.'' And, lastly, I will ask your attention to two small books which have been published within the past two or three years. (These books may be found at the Buffalo Public Library). The first book has this title, ''Perse Playbooks. No. 1. Dramatic Work by Boys of The Perse School, Cambridge.'' (England). The second, ''The Eurhythmics of Jaques-Dalcroze.''

In the first book are three playlets, written by the boys of the school, none of whom are over fifteen years of age, and some introductory remarks by the officials of the school. The Headmaster says: ''Acting is one of the most potent means of learning. Thought, word and act, linked together make an impression such as nothing else can. In this direction lies the salvation of our schools. We all know how dull a text-book is; a history of England, a manual of grammer, even chemistry books are sometimes dull. But, if the teacher uses his book as a suggestion, makes his history a story, sets his pupils to act it in make-believe, before they know what they are doing they are practising English composition and English grammer, and learning English history.

''The plays in this volume are specimens of the result. From improvised scenes, with the teacher's help to guide them, they have gradually perfected their play, so that it is the product of united effort. I think it shows a natural and unforced spirit which will convince the intelligent reader that the pupils have been taught, not crammed; they have learnt to do, to express themselves, not to reproduce some one else's opinion in worse language than they were originally put in.''

The Master of the Players says: ''It is not acting we teach the boys, but the value of action. If it be said that boys are born play-actors we do not agree, but, to use the words in a wider and more natural sense, everyone knows that boys love to play and live to be active.

"Proficiency does not come from reading and listening, but from action, from doing and from experience.

"Good work is more often the result of spontaneous effort and free interest than of compulsion and forced application.

"The natural means of study in youth is play."

Several years ago Professor Jaques-Dalcroze established a school for music at Hellerau, in Saxony, four miles outside of the city of Dresden. (With the outbreak of the present war the Professor became **persona non grata** to the German Government and the school was removed to Switzerland, I think). In this school the teaching is through a combination of music and gymnastics; that is, music is taught through the actualization of rhythm, the brain is brought to a realization of music through body actions associated with the music-idea.

The Professor says: "The object of the method is, in the first instance, to create by the help of rhythm a rapid and regular current of communication between brain and body; and what differentiates my physical exercises from those of present-day methods of muscular development is that each of them is conceived in the form which can most quickly establish in the brain the image of the movement studied. : : : : The creation in the organism of a rapid and easy means of communication between thought and its means of expression by movements allows the personality free play, giving it character, strength and life to an extraordinary degree. : : : : Lessons in rhythmic gymnastics help children in their other lessons, for they develope the powers of observation, of analyzing, of understanding and of memory, thus making them orderly and precise. : : : : From many years experience of music teaching I have gradually produced a method which gives the child experiences instead of musical knowledge."

I have quoted at some length from these books, but the matter quoted is important as it helps to confirm what I have said in regard to action and development. In the first book we are shown how the child learns grace of tongue and of thought through action, action that holds his interest; in the second how he acquires grace of body and of expression in the same manner. At the Perse school they start with an idea; the brain has something upon which to fix its interest, and upon which it can build: the memory is built up gradually, each successive fact in the memory being connected with the one behind and the one before in a logical and interesting manner. Professor Dalcroze also developes the memory in a logical manner. With his rhythmic movements he associates with the music-idea something that fixes the latter in the cortical cells. He says that he gives experience instead of knowledge. But experience **is** knowledge. What he means is that he gives experience-ideas upon which to build knowledge. It is experience that teaches, and the manner in which the experience is acquired determines the value of the teaching.

Thought is a function of the mind; one might say, with truth, that thought is mind. Thought begins early, in the child before it is obvious to the observer, but in the beginning it is of little value as thought. It is merely reflex action. The child's brain cells attempt the exercise of the function which belongs to them, but, lacking strength and experience, they cannot think properly. The child has neither the ideas nor the images upon which to base right thought. He thinks according to his own experiences, and his own experiences being very limited or imperfect, his thought takes on a bias. His association of mind-images becomes improperly adjusted. His thought sequence is faulty.

The people whom I have quoted have seen the results of action in connection with brain development, but they do not quite appreciate what it means. They see that the individual is better because of action, but they do not know why he is better. They must understand that it is action that developes the brain cells, and that wrong action will develope them as well as right action. Knowing this it becomes perfectly obvious to anyone that the action must be the right action in the beginning. But, the man brain must not be put to work too early, for its development must not be forced. The animal brain requires a certain period for its development, that period lengthening as we ascend the scale. Man, the white man, requires the longest period of all the animals. If we force the brain we shorten this development period; we bring out the function of the centres before the centres are ready for work, thereby weakening the cells and shortening their life period.

Before ending this paper let us return to the school room again, for a moment. I said that the modern school room should be eliminated. I re-affirm that; and I will go further: I would completely alter the methods of that room.

The first things to be considered would be the methods of study and recitation. A friend, with whom I have discussed this matter in a general way, gave me a valuable hint. He said: "I am beginning to think that the recitation hour should be the study hour." I am inclined to agree with this. The object of education is the permanent registration of useful ideas; and, in order to make the registration permanent, the method of registration must be the right one. Under the present method the pupil does the greater portion of his studying under the direction of either his parents or himself. In either case he has a poor teacher. If he has to work out the matter for himself he is just as apt to go wrong as right; and, if his parents take a hand in the process, he actually learns little or nothing. The system of today prepares the pupil for the recitation hour but not for the future; it is a temporary make-shift, it does not accomplish a permanent registration of ideas. It would be better, for the beginner at least, if the recitation hour were used in showing the pupil how to proceed; if he were drilled in the method of procedure until he had learned how for himself, until his consciousness had recognized it. It must not become merely automatic, that is, a reflex action, for then the pupil is not conscious of what he is doing. No pupil has learned a fact until he is conscious of that fact, until he can recall it to active consciousness. If he cannot recall it of his own volition his cells have only a reflex knowledge of it, and the knowledge is of no practical use to him. Now, if the pupil can be taught in the right way, if he can be guided into the permanent way, his foundation ideas will be laid in the proper manner, and the registration will be permanent. If the lessons were discussed in open class, if the teacher put the subjects before the pupil in a live, in a graphic manner, they would be learned in the easiest possible manner, without effort and, consequently, without fatigue. Learned in this manner the idea would be registered for life, not merely for the registration hour.

Another change would concern the teacher. I would have the teacher eliminate all personal opinions, all intuitive ideas. Many crimes are committed in the name of intuition, for, intuition is only a sort of subconscious guessing. The school room is no place for guessing. If a teacher has ideas let them be presented to a committee maintained for that purpose, and let the committee work them out. I want to quote again from Professor Dalcroze. In regard to the teacher he says: "It is quite impossible to develope others until one has proved one's own powers in every direction, until one has learnt to conquer oneself, to make oneself better, to suppress bad tendencies, to strengthen good ones, and, in the place of the primitive being, to make one more complete who, having consciously formed oneself, knows his powers. Only in proportion as one developes oneself is one able to help others to develope."

In the new school room the word "punishment" will be almost unknown, for, the occasions for punishment will be very rare. But, if an occasion does arise the naming of the punishment will not be left with the teacher. There will be a punishment committee, on which will be some of the pupils themselves, to decide all such matters.

Why do I object to the teacher being the one to measure justice? Because she occupies the position of an autocrat, and autocrats often go wrong; she is as apt to administer injustice as she is justice. Her administration is, often, only guessing. Here is a case in point. I know very well a boy of nine who attends the public school. One day a substitute teacher took charge of his room. During the morning she had occasion to leave the room for five minutes, and while she was away there was considerable disorder in the room. She was near enough to hear the noise, and when she returned she looked about for the guilty ones. She asked who had caused the disorder, and getting no reply, hit upon my boy friend as the one most to blame, although he had had no hand in the disturbance. In spite of his denial she adjudged him guilty and ordered him to stand in a corner of the room for one hour.

Now, there was an occasion where the punishment idea was the wrong one, from whichever angle it might be viewed. First: The teacher did wrong in doubting the boy's word. If one doubts a boy he becomes careless as to your opinion of him. When the teacher is unfair the boy knows it, and his first thought is to get even. He fights fire with fire.

Second: The teacher did wrong in punishing the boy for something he did not do. It goes without saying that punishment under such conditions is wrong, but still, we do have to say it. It may be claimed that, this being a substitute teacher, there was some excuse for it; but, **there is no excuse for injustice in the school room.** A substitute teacher is a teacher, subject to all the laws governing teachers. What she did might be done by any teacher.

Finally: The teacher did wrong in punishing at all, and in administering the punishment she did. The punishment was totally without reason; it was, in fact, criminal. Think of making a boy of nine, a boy whose muscles cannot keep quiet, stand completely still for one whole hour. Did the reader ever try to stand **still** for an hour? And was it easy? **Then what about that boy?** That restless, growing, and, at the time, insulted boy? Surely that punishment **was** a crime, a crime against reason and against boy nature. In committing crimes we go backward instead of forward.

The assertion is often made that civilization is a failure; but that assertion is false. The methods of civilization often are wrong, but its principles cannot be. If there is any failure it is because we lack the proper method for the development of the individual. Man is still in the animal stage; he lacks the degree of development necessary for the appreciation of the finer principles of civilization. The real lack here is the lack of brain development. What is needed is a system that will take the child brain and develope it according to the laws of development as revealed in the evolutionary history of the brain. A brain so developed will be a brain in complete mastery of itself; it will be a brain having full self-consciousness, a brain that understands, a brain that is aware of its own powers.

143 Allen Street.

